# Influence of Wood Chemical Composition on Liquefaction Efficiency and Polyurethane Foam Properties: A Study of Red Angico and Mahogany

**DOI:** 10.3390/ma19020417

**Published:** 2026-01-21

**Authors:** Emilly Silva, Luísa Cruz-Lopes, Idalina Domingos, Fabricio Gonçalves, Bruna da Silva Cruz, Michelângelo Fassarella, Antônio Thiago de Almeida, Bruno Esteves

**Affiliations:** 1 Department of Forest and Wood Sciences, Federal University of Espírito Santo, Jerônimo Monteiro 29550-000, ES, Brazil; emillysoaresgomes@gmail.com (E.S.); fabricio.goncalves@ufes.br (F.G.); brunacruzufes@gmail.com (B.d.S.C.); michelangelo.fassarella@gmail.com (M.F.); thiagosoares456@hotmail.com (A.T.d.A.); 2 CERNAS (Centre for Natural Resources, Environment and Society), Research Centre, Polytechnic University of Viseu, Av. Cor. José Maria Vale de Andrade, 3504-510 Viseu, Portugal; ijd@estgv.ipv.pt (I.D.); bruno@estgv.ipv.pt (B.E.)

**Keywords:** bio-based polyols, hydroxyl value, lignocellulosic biomass, liquefaction, mechanical properties, polyurethane foams

## Abstract

Biomass liquefaction is a thermochemical process that converts lignocellulosic materials into reactive liquid intermediates, enabling the production of bio-based polyols as a sustainable alternative to petroleum-derived chemicals. This study investigates the liquefaction of two lignocellulosic biomasses, Red Angico (*Anadenanthera colubrina*) and Mahogany (*Swietenia macrophylla*), using a glycerol–ethylene glycol polyalcohol system, chosen for its renewable origin and high solvating efficiency. The resulting polyols were used to produce polyurethane (PU) foams, and their properties were evaluated in relation to biomass composition. The chemical composition of each biomass significantly influenced its liquefaction behavior and polyol characteristics. Mahogany achieved higher liquefaction efficiency, whereas Red Angico polyols generated PU foams with superior mechanical performance, highlighting the influence of species-specific chemistry. Water content and isocyanate index were found to modulate foam structure and compressive strength. This work demonstrates how tailored liquefaction strategies using polyalcohol systems can optimize bio-based PU foam properties, providing a sustainable route for high-performance polymer materials.

## 1. Introduction

Red Angico (*Anadenanthera colubrina*) and Mahogany (*Swietenia macrophylla*) are two representative hardwood species native to tropical and subtropical regions of the Americas, with a strong presence in Brazil. Red Angico, locally known as *Angico Vermelho*, is a native species distributed across the Northeast, Central-West, and Southeast regions of Brazil, occurring in diverse phytogeographic domains such as the Caatinga, Cerrado, and Atlantic Forest [1,2,3,4]. It is valued for its dense and durable wood, as well as for its traditional medicinal applications [5]. Mahogany (*Swietenia macrophylla*), regarded as the original mahogany species, naturally occurs in transitional evergreen forests, particularly in southeastern Pará, Brazil [6]. Owing to its aesthetic quality and mechanical performance, mahogany is among the most valuable tropical timbers worldwide, widely used in high-end furniture and musical instruments. Both species also play important ecological roles and are subject to conservation concerns due to historical overexploitation.

Previous studies have demonstrated that the chemical composition of *Swietenia macrophylla* wood varies considerably depending on geographic origin, environmental conditions, and analytical methodology. Reported values of cellulose, hemicellulose, and lignin differ markedly among samples from Asia and South America, indicating strong site-dependent variability [7,8]. In addition, mahogany wood contains significant amounts of extractives and phytochemicals, including phenolic compounds such as flavonoids and tannins, which contribute to its biological activity and may influence thermochemical conversion processes [9]. In particular, extractive contents in *Swietenia* species range widely, from values below 4% to more than 28%, depending on tree age, wood position, solvent type, and regional factors [10,11,12]. This variability is highly relevant for biomass liquefaction, as extractives can affect reaction efficiency, polyol composition, and subsequent polymer performance.

In contrast, the chemical composition of Red Angico (*Anadenanthera colubrina*) has been far less explored in the literature. Available studies indicate lignin contents ranging from approximately 16% to 25%, depending on geographic origin and forest management conditions, along with substantial amounts of structural polysaccharides and extractives [13,14]. These characteristics suggest that Red Angico represents a promising, yet underutilized, lignocellulosic feedstock for thermochemical conversion processes. However, systematic investigations linking its chemical composition to liquefaction behavior and polymer applications remain scarce.

Liquefaction is a well-established route for converting lignocellulosic biomass into reactive liquid intermediates suitable for the production of bio-based polymers [15,16]. During wood liquefaction, hemicelluloses are affected first because they are the least thermally stable and more susceptible to acid or heat-induced cleavage, followed by cellulose, which undergoes partial depolymerization due to its crystalline structure, while lignin is broken down last, as its complex aromatic network is more resistant, but eventually forms soluble phenolic fragments that contribute hydroxyl groups for polyol formation [17]. Common liquefaction agents include phenol and polyhydric alcohols, typically used in the presence of acid catalysts, as well as alternative solvents such as cyclic carbonates, ionic liquids, and dibasic esters [18,19]. Polyhydric alcohol liquefaction, in particular, has attracted attention due to its versatility in producing bio-based polyols for epoxy resins and polyurethane systems [20]. The efficiency of the liquefaction process is strongly influenced by operational parameters such as temperature, reaction time, catalyst loading, and solvent-to-biomass ratio, while post-treatment steps, including acid neutralization, are critical to ensure suitable reactivity toward isocyanates in polyurethane formulations [21,22].

Polyurethane (PU) foam formation involves an isocyanate, polyol, blowing agent, catalyst, and surfactant, each contributing to the reactions that govern foam development. Rigid PU foams are commonly produced using aromatic isocyanates such as toluene diisocyanate and polymeric diphenylmethane diisocyanate (MDI) [23]. Catalysts typically include tertiary amines and organometallic compounds; the former mainly catalyze the isocyanate–water reaction, while the latter promote urethane formation between isocyanates and polyols [23,24]. Foam expansion is achieved using physical blowing agents, such as n-pentane, or chemical blowing agents, most notably water, which generates carbon dioxide through reaction with isocyanates [23]. The choice of formulation components strongly influences foam cell structure and, consequently, its mechanical, thermal, and acoustic properties [25]. Surfactants, most commonly silicone-based, ensure proper mixing, stabilize the cellular structure, and prevent cell collapse during foaming [23].

Despite the growing body of literature on biomass liquefaction, comparative studies evaluating tropical hardwood species with distinct chemical profiles under similar processing conditions remain limited. Moreover, the direct relationship between wood composition, liquefaction behavior, and the performance of polyurethane foams is still insufficiently understood, particularly for native Brazilian species.

In this context, the present study aims to investigate the chemical composition and liquefaction behavior of Red Angico (*Anadenanthera colubrina*) and Mahogany (*Swietenia macrophylla*) and to evaluate the properties of polyurethane foams produced from the resulting bio-based polyols. The novelty of this work lies in the comparative experimental design, which systematically relates differences in lignocellulosic composition to liquefaction efficiency, polyol characteristics, and foam performance. By directly comparing two tropical hardwood species with contrasting chemical profiles, this study provides new insights into the suitability of underexplored native biomasses for sustainable polyurethane applications.

## 2. Materials and Methods

### 2.1. Materials

Red Angico (*Anadenanthera colubrina*) and Mahogany (*Swietenia macrophylla*) wood samples were sourced from managed forest reserves in the southeastern region of Brazil. Upon collection, the samples were air-dried to constant weight, ground using a Wiley-type mill (Thomas Scientific, Swedesboro, NJ, USA), and classified by particle size through standard sieving using a vibratory sieve shaker (Vibratory Sieve Shaker AS 200, Retsch GmbH, Haan, Germany) for 30 min at 50 rpm. The sieved material was divided into three granulometric fractions: >40 mesh (>0.450 mm), 40–60 mesh (0.250–0.425 mm) and <60 mesh (<0.250 mm). Prior to any analysis, all fractions were oven-dried at 100 °C for 24 h. Unless stated otherwise, the 40–60 mesh fraction was selected for chemical characterization and liquefaction experiments. All reagents used throughout the procedures were of analytical grade.

### 2.2. Chemical Characterization

The 40–60 mesh wood fractions of Red Angico and Mahogany were analyzed for their chemical composition, including ash, extractives (organic solvents and water), lignin (acid-insoluble and soluble), α-cellulose, and hemicelluloses. Samples were conditioned by oven-drying at 105 °C for a minimum of 24 h prior to analysis. All analyses were performed in triplicate according to the Technical Association of the Pulp and Paper Industry (TAPPI) standards.

Ash content was quantified via calcination at 525 °C following the TAPPI T 211 om-93 method [26]. Sequential Soxhlet extraction was employed for determining extractives, using 150 mL each of dichloromethane, ethanol, and hot distilled water, as described in TAPPI T 204 om-88 [27]. Each solvent extraction was performed for 6 h (dichloromethane) and 16 h (ethanol and water), using 10 g of oven-dried sample per test.

Lignin quantification was carried out using a modified Klason procedure. Initially, 72% H_2_SO_4_ hydrolysis was conducted at 30 °C for 1 h in a water bath, followed by secondary hydrolysis with 3% H_2_SO_4_ at 120 °C in an autoclave for 1 h. The resulting acid-insoluble residue was filtered through a G2-grade sintered glass crucible, washed with warm water and acetone, and dried to constant weight at 100 °C. The percentage of Klason lignin was calculated using the following formula:

(1) $$Lignin\left(\%\right)=\frac{Insoluble\;residue}{Dried\;material}\times 100$$

Soluble lignin content was determined spectrophotometrically by absorbance at 205 nm.

The holocellulose content of Red Angico and Mahogany samples was determined using the sodium chlorite–acetic acid delignification method, adapted from the procedure described by Wise et al. [28]. Approximately 2.00 ± 0.01 g of extractive-free, oven-dried wood material (40–60 mesh fraction) was placed in a 250 mL Erlenmeyer flask. To this, 100 mL of distilled water was added, followed by 0.3 g of sodium chlorite (NaClO_2_) and 0.2 mL of glacial acetic acid. The flask was sealed with a loose-fitting stopper and placed in a water bath at 70 °C. Every 1 h, an additional 0.3 g of sodium chlorite and 0.2 mL of acetic acid were added, and the reaction was allowed to proceed for a total of 4 h, with occasional swirling to ensure even contact.

Upon completion of the delignification, the mixture was cooled to room temperature. The solid residue was filtered using a G2 sintered glass crucible and washed thoroughly with cold distilled water until the filtrate was neutral (pH ~ 7). The retained material, composed of both cellulose and hemicelluloses, was then oven-dried at 105 °C until constant weight.

The holocellulose content was calculated based on the dry weight of the residue relative to the initial dry weight of the sample:

(2) $$Holocellulose\left(\%\right)=\frac{Dry\;weight\;of\;insoluble\;residue}{Initial\;dry\;weight\;of\;sample}\times 100$$

All determinations were carried out in triplicate to ensure reproducibility.

The α-cellulose content of Red Angico and Mahogany samples was determined following standard alkaline extraction procedures adapted from TAPPI T 429 cm-23 [29]. For each determination, 1.00 ± 0.01 g of holocellulose was treated with 17.5% sodium hydroxide (NaOH) solution at room temperature. The sample was stirred gently in the NaOH solution for 30 min, promoting the dissolution of hemicelluloses and β-/γ-cellulose fractions. The suspension was then diluted with distilled water to reduce the NaOH concentration to approximately 8.3% and allowed to stand for 1 h. The insoluble α-cellulose was filtered using a G2 sintered glass crucible, washed thoroughly with 1% acetic acid followed by multiple rinses with hot distilled water until the filtrate reached neutral pH. The residue was dried in an oven at 105 °C to constant weight.

The α-cellulose content was calculated as:

(3) $$\alpha -Cellulose\left(\%\right)=\frac{Dry\;weight\;of\;insoluble\;residue}{Initial\;dry\;weight\;of\;sample}\times 100$$

All measurements were performed in triplicate for accuracy.

Hemicellulose content was estimated by subtracting the values of Holocellulose and α-cellulose.

### 2.3. Liquefaction

Liquefaction trials were designed to investigate the influence of temperature and reaction time on the solvolysis of Red Angico and Mahogany wood. Experiments were conducted in a 600 mL double-jacketed stainless-steel reactor (Parr Instrument Company, Moline, IL, USA) equipped with mechanical stirring and temperature control, heated externally with a thermostatic oil bath. Each liquefaction mixture contained 10 g of dried wood particles (40–60 mesh), 100 mL of a 1:1 (*v*/*v*) blend of glycerol (87%) and ethylene glycol as the solvent system, and 3% (*w*/*w*) sulfuric acid as a catalyst. The reaction conditions were varied as follows: fixed temperature at 180 °C, with reaction times of 15, 30, and 60 min and fixed reaction time of 60 min, with temperatures of 140 °C, 160 °C, and 180 °C.

Upon completion, the reaction mixtures were rapidly cooled to room temperature. The liquefied products were diluted with methanol and vacuum-filtered to separate unreacted residues. The efficiency of liquefaction was assessed based on the mass of residual solids and the solubility of the filtrate.

### 2.4. Determination of Hydroxyl Value (OH)

The OH index was determined based on methodologies described in the literature, which involve the potentiometric titration of the residual acetic acid after the esterification of the free OH groups [30,31,32]. Approximately 20 mg of the polyol from each species were placed in test tubes, followed by the addition of 0.1 mL of an acetylating mixture prepared by combining 2.35 mL of acetic anhydride solution with 2 mL of pyridine. The contents of the tubes were homogenized and kept in an oven at 50 ± 2 °C for 24 h. After cooling, 10 mL of acetone and 10 mL of distilled water were added to remove residual reagents. The mixture was then titrated with standardized 0.1 N lithium hydroxide (LiOH), and the OH index was determined according to Equations (4) and (5).

(4) $$\mathrm{OH}\left(\%\right)=\frac{\left[\left(\mathrm{ms}\;\times \;\frac{\mathrm{vb}}{\mathrm{mb}}\right)-v\right]\times \;f\;\times \;1.7\;\times \;100}{w}$$

(5) $$\mathrm{IOH}\left(\mathrm{mg}\frac{\mathrm{KOH}}{g}\right)=33\;\times \;\mathrm{OH}\left(\%\right)$$

where V is the volume of LiOH solution required for the titration of the sample (mL); Vb is the volume of LiOH solution required for the titration of the blank (mL); ms is the acetylating mixture of the sample (mg); mb is the blank (acetic anhydride and pyridine) in mg; f is the standardized titer of LiOH solution; W is the weight of the sample (mg); and 1.7 is the mass, in mg, of OH groups equivalent to 1 mL of 0.1 M LiOH.

### 2.5. Polyurethane Foams Production

Polyurethane (PU) foams were produced using polyols obtained from liquefied Mahogany and Red Angico woods at 180 °C and 60 min. The polyols were neutralized with NaOH and dried before been used for the production of foams.

Polymeric methylene diphenyl diisocyanate (pMDI), with a 31% NCO content and 2.7 functionality, was used as the isocyanate source. Water served as the chemical blowing agent, reacting with the isocyanate to produce carbon dioxide, which promoted foam expansion. A silicone-based surfactant (Tegostab) was incorporated to stabilize the foam structure and ensure uniform cell morphology throughout the foaming process. No additional catalysts were added to the formulation (Table 1).

Foam formulations were prepared by varying both the isocyanate index and the water content. The isocyanate index was adjusted from 1.0 to 4.9, based on the OH number of the liquefied wood polyols, to investigate the effect of crosslink density on foam properties. Water content was 5%, 7.5% and 10% relative to the total polyol weight, to evaluate the influence of gas generation on foam structure and mechanical performance.

In each experiment, the liquefied wood polyols, water, and Tegostab surfactant were first mixed at 1500 rpm for 1 min using a high-speed mechanical stirrer to achieve a homogeneous mixture. Subsequently, the calculated amount of pMDI was added, and the mixture was stirred for an additional 1 min at the same speed. The reactive mixture was then immediately poured into open cylindrical molds with a diameter of 35 mm and allowed to freely expand at room temperature. After full expansion, the foams were cured at ambient conditions for 48 h before testing. A 35 mm high cylindrical shape sample was cut from the mold ensuring there was no empty spaces in the foam.

The resulting PU foams were characterized in terms of their apparent density, compressive strength, and compressive modulus. The apparent density was determined according to ASTM D1622 [33], by measuring the mass and volume of the cured foam samples. Compressive strength and compressive modulus were measured following ASTM D1621 [34] using a Servosis I-405/5 universal testing machine (Servosis, Madrid, Spain). All measurements were performed in triplicate, and the reported values represent the average of these measurements.

**Figure 1:**
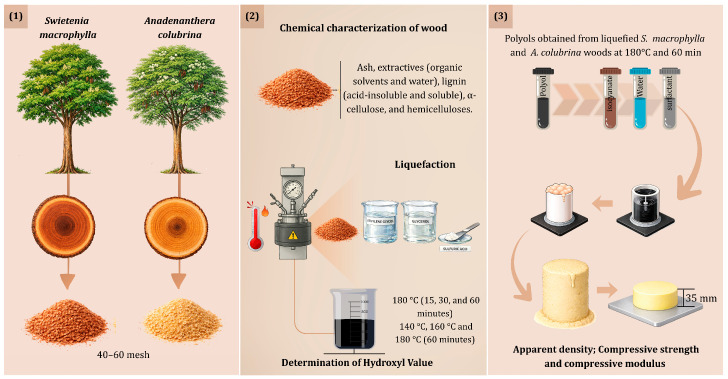
Schematic representation of the methodology for wood characterization, liquefaction process, and production of polyurethane foams from liquefied polyols.

In order to facilitate the understanding of the methodology adopted for foam production, Figure 1 schematically illustrates the steps of preparing the raw material for wood characterization (1), the liquefaction process (2), and the generation of foams from the liquefied polyols (3).

## 3. Results and Discussion

The chemical composition results obtained in this study were compared with previously reported data for Red Angico wood in order to contextualize the observed values and assess the influence of biomass variability (Table 2). Regarding extractives, the present study reports fractions soluble in dichloromethane of 0.22%, soluble in ethanol of 3.25%, and soluble in hot water of 1.77%, resulting in a total extractive content of 5.24%. This value is lower than those commonly reported in the literature for this species and for wood samples obtained from managed forests in northeastern Brazil [13,14]. These differences may be attributed to variations in biomass origin, extraction methodology, solvent polarity, and physiological characteristics of the sampled trees, highlighting the inherent variability of lignocellulosic materials.

Regarding lignin content, the Klason lignin value determined in this study (20.63%) is consistent with previously reported data, closely aligning with the 18.8% reported in the literature, falling within the broader range observed in regional studies, which varies from 16.37% to 24.89% [4,13]. This agreement confirms that the Red Angico biomass used in the present work is representative of the species and supports its classification as a hard-wood with moderate to high lignin content, which is relevant for interpreting its liquefaction behavior and subsequent performance in polyurethane foam production.

The α-cellulose content obtained in this study was 48.44%, indicating a relatively high proportion of purified cellulose in the Red Angico biomass. Considering that glucan is commonly used as an indirect estimate of cellulose content in compositional analyses, the elevated α-cellulose value observed here may suggest a higher degree of fiber purity in the analyzed samples. This difference may also be associated with methodological aspects, since α-cellulose determination selectively isolates the pure cellulose fraction, excluding other polysaccharides [13].

The hemicellulose content observed in this study was 25.68%, indicating a relatively high proportion of structural polysaccharides in the Red Angico biomass. When compared with previous reports, this value is higher than those commonly described for this species, which may be related to differences in analytical approaches, particularly regarding the quantification of individual sugar components and the possible contribution of galactans and uronic acids to the hemicellulosic fraction [13]. Overall, the data obtained in this study are consistent with previously published trends for Red Angico wood, especially regarding lignin content, while indicating a higher proportion of structural polysaccharides (cellulose and hemicelluloses) and a lower extractive content. These differences highlight the influence of geographic origin, forest management practices, and analytical methodologies on the reported chemical composition of this species.

The chemical composition of Mahogany varies widely across different studies and regions, as observed when comparing the results obtained in this study with previously reported data (Table 2). The α-cellulose content measured here was 18.24%, indicating a relatively low proportion of purified cellulose in the analyzed biomass. In contrast, the hemicellulose content reached 56.11%, suggesting an unusually high proportion of structural polysaccharides, which may be associated with differences in sample preparation, analytical methodology, or botanical and environmental factors [35].

The Klason lignin content determined in this study was 19.51%, which is consistent with values commonly reported for Mahogany wood, depending on geographic origin and growing conditions [8,36,37]. Regarding extractives, the contents soluble in dichloromethane (0.42%), ethanol (4.02%), and hot water (1.70%) resulted in a total extractive content of 6.14%, indicating a relatively low proportion of extractives compared to values frequently described in the literature [10,11,38]. Overall, the results indicate a Mahogany sample characterized by high hemicellulose content, low cellulose content, and relatively low extractives. These differences highlight the strong influence of geographic origin, environmental conditions, tree physiology, and analytical methodologies on the reported chemical composition of Mahogany wood [12].

The comparative chemical composition of Red Angico and Mahogany presented in Table 2 reveals notable differences in their primary and extractive constituents, which may be indicative of their respective structural and functional adaptations. Both species were analyzed for extractives in dichloromethane, ethanol, and hot water, as well as for major lignocellulosic components—namely, Klason lignin, α-cellulose, and hemicelluloses. Red Angico exhibited a lower content of dichloromethane-soluble extractives (0.22%) compared to Mahogany (0.42%), suggesting a relatively lower concentration of non-polar or lipophilic compounds such as waxes, fats, or resin acids. Similarly, the ethanol extractives were higher in Mahogany (4.02%) than in Red Angico (3.25%), indicating a greater abundance of polar extractives such as phenolics and low-molecular-weight sugars in Mahogany. In contrast, the hot water-soluble fraction was nearly identical between the two species, with Red Angico at 1.77% and Mahogany at 1.70%, reflecting a comparable solubility of water-soluble polysaccharides or other hydrophilic substances.

A more substantial difference is observed in the lignocellulosic matrix. Red Angico has a higher α-cellulose content (48.44%) than Mahogany (18.24%), indicating a greater proportion of crystalline cellulose, which is crucial for mechanical strength and resistance to biodegradation. Conversely, Mahogany demonstrates a remarkably higher hemicellulose content (56.11%) compared to Red Angico (25.68%). The total Klason lignin content is slightly higher in Red Angico (20.63%) than in Mahogany (19.51%), though the difference is marginal.

**Figure 2:**
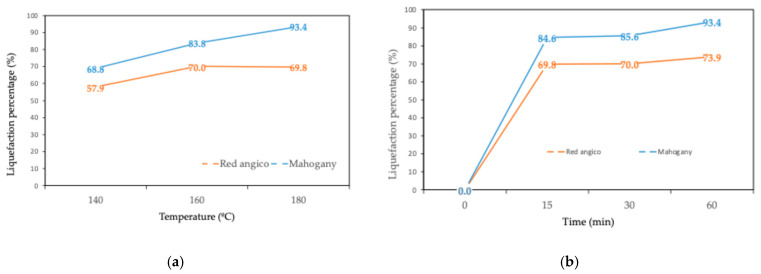
(a) Variation in liquefaction percentage with temperature for liquefaction during 60 min; (b) variation in liquefaction percentage with time for liquefaction at 180 °C.

The liquefaction behavior of Red angico and Mahogany wood using polyalcohol liquefaction at a constant temperature of 180 °C across varying reaction times, and at a fixed reaction time of 60 min across different temperatures are presented in Figure 2a,b. The results demonstrate clear distinctions in the reactivity and thermal behavior of the two wood species under the tested conditions. At 180 °C, both species exhibited rapid liquefaction within the first 15 min. Red angico reached a liquefaction percentage of 69.8%, while Mahogany achieved a significantly higher value of 84.6%, indicating greater susceptibility of Mahogany to chemical breakdown in the early stages of the reaction. This is most likely due to the higher hemicellulose content of Mahogany as seen in Table 2. Hemicelluloses are known to be the most susceptible polymers to hydrolysis [39]. As the reaction proceeded to 30 min, the liquefaction efficiency of Red Angico increased slightly to 70.0%, while Mahogany rose to 85.6%. By 60 min, Mahogany reached a liquefaction level of 93.4%, suggesting near-complete conversion, whereas Red Angico attained 73.9%, reflecting a slower or more limited reactivity under the same conditions. Red Angico exhibits a slower liquefaction rate due to its high cellulose and lignin content, making it more resistant to acid hydrolysis and requiring longer times or harsher conditions; its lower hemicellulose content results in a less easily degradable carbohydrate fraction initially, leading to a steady but slower liquefaction process with lower yields at shorter time intervals.

Temperature-dependent liquefaction at a fixed reaction time of 60 min revealed marked differences in the thermal behavior of the two wood species. At 140 °C, Red Angico and Mahogany exhibited liquefaction yields of 57.9% and 68.8%, respectively, indicating a higher initial susceptibility of Mahogany to polyalcohol-assisted thermal depolymerization. When the temperature was increased to 160 °C, liquefaction efficiency improved for both biomasses, reaching 70.0% for Red Angico and 83.8% for Mahogany, confirming the strong influence of thermal energy on biomass solubilization (Figure 2a). At 180 °C, Mahogany reached a liquefaction yield of 93.4%, demonstrating a continuous enhancement of conversion with temperature, whereas Red Angico showed only a slight increase to 73.9%, suggesting that its structure becomes less responsive to further thermal intensification. This behavior indicates that Mahogany presents a chemical composition and structural organization more favorable to polyalcohol penetration and bond cleavage, likely due to differences in lignin architecture and carbohydrate accessibility [40,41,42].

In contrast, Red Angico appears to reach its optimal conversion efficiency around 160 °C, which may be associated with a more condensed lignin network or a higher resistance to thermal depolymerization. These results highlight the strong species dependence of liquefaction performance and demonstrate that process conditions must be specifically optimized for each biomass in order to maximize conversion efficiency and product yield.

The OH index of polyols derived from the liquefaction of Mahogany and Red Angico using a glycerol and ethylene glycol cosolvent system illustrates the dynamic chemical changes occurring during thermal processing of lignocellulosic biomass (Figure 2). Liquefaction breaks down the complex structure of biomass, composed primarily of cellulose, hemicellulose, and lignin, into smaller, reactive fragments. This initial depolymerization exposes or generates free OH groups, which typically results in an increase in the OH index, a key parameter reflecting the number of reactive sites available for further chemical modification, such as polyurethane synthesis.

The OH index trends observed for Mahogany and Red Angico during liquefaction at 180 °C reflect distinct differences in their chemical behavior under acidic conditions. Mahogany shows a continuous and significant increase in OH index, from approximately 580 at 15 min to over 1200 at 60 min (Figure 2b). This steady rise indicates effective and ongoing depolymerization, primarily driven by its high hemicellulose content (56.11%), which is readily hydrolyzed in the presence of sulfuric acid. The continued increase beyond 30 min suggests that both hemicellulose and possibly some cellulose fractions are being converted into OH-rich products, contributing to a higher functional group density in the liquefied material. In contrast, Red Angico exhibits a declining OH index over the same time range, but starting from about 1120 at 15 min and decreasing then to around 620 at 60 min. The decreasing OH index over time implies that recondensation or cross-linking reactions may dominate as the reaction proceeds, leading to the consumption of free OH groups and a corresponding drop in the measured index.

**Figure 3:**
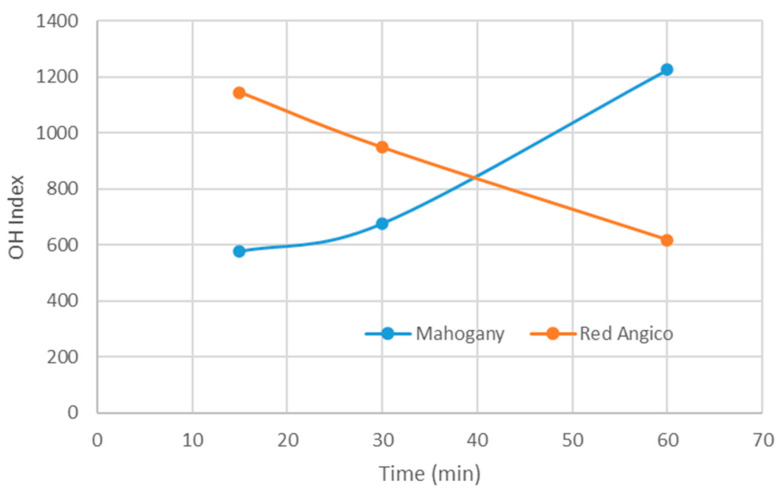
Variation in OH index as a function of liquefaction time for polyols derived from Mahogany and Red Angico liquefied at 180 °C.

Overall, the data indicate that Mahogany needs more time to achieve higher OH values (Figure 3). In contrast, Red Angico, while initially reactive, may be prone to secondary reactions that reduce its functional group availability, suggesting a need for shorter reaction times or modified conditions to preserve its OH content. This decline is consistent with the occurrence of secondary reactions such as condensation, dehydration, and thermal degradation. These processes can consume OH groups, form water, or convert hydroxyls into other functional groups, thus reducing the overall OH content. Similar results were presented before were increased reaction time lead to a decrease in OH value [43]. As the liquefaction time increased, the OH value of the polyol from wheat-straw lignocellulose with steam-explosion pretreatment decreases.

A similar effect has been reported for higher temperatures. Previous studies have shown that increasing the reaction temperature from 130 °C to 190 °C leads to a decrease in the OH number of biopolyols, as observed in the liquefaction of empty fruit bunch lignin residues and in related systems [44,45].

These contrasting trends highlight the complex interaction of depolymerization and re-polymerization mechanisms during liquefaction. While early stages typically enhance the OH index due to fragmentation and exposure of new OH sites, prolonged reaction times often lead to a decline in OH content due to increased molecular weight through polycondensation and loss of hydroxyls. This species-specific behavior is crucial for tailoring liquefaction conditions to optimize the chemical functionality of resulting polyols for applications such as bio-based resins and foams.

**Figure 4:**
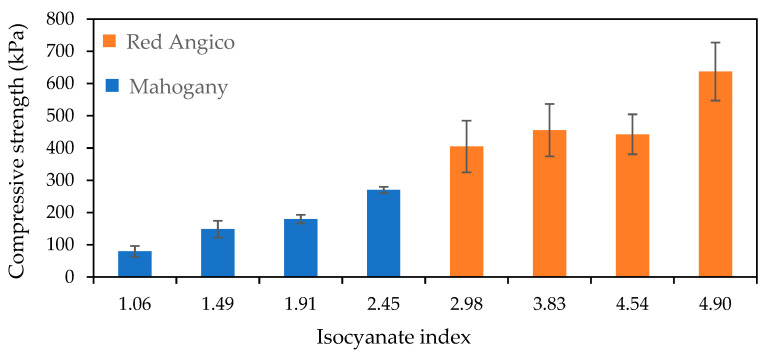
Variation in Compressive strength with Isocyanate index for polyurethane foams derived from for Mahogany (blue) and Red Angico (Orange). Error bars represent the standard deviation.

The compressive properties of foams synthesized from liquefied Red Angico wood demonstrated a clear dependency on the isocyanate index, as illustrated in Figure 4. The compressive strength increased with rising isocyanate index, suggesting an enhancement in the mechanical integrity of the polymer matrix.

The evolution of compressive strength with increasing isocyanate index reflects the progressive development of a more highly crosslinked polyurethane network. At an isocyanate index of 1.06, the Mahogany-based foams exhibited a compressive strength of approximately 80 kPa, indicating limited network formation and weak structural cohesion. As the isocyanate index increased to 1.49 and 1.92, the compressive strength rose to about 150 kPa and 180 kPa, respectively, demonstrating that higher NCO availability promotes more extensive urethane and urea bond formation, resulting in improved mechanical resistance. At the highest isocyanate index of 2.45, the compressive strength reached approximately 270 kPa, confirming that a greater degree of crosslinking leads to a more rigid and load-bearing foam structure. For Red Angico foams, a similar trend was observed, but with substantially higher mechanical performance. At an isocyanate index of 2.98, the compressive strength was approximately 400 kPa, increasing to about 460 kPa with further NCO enrichment. At the highest isocyanate index of 4.90, the compressive strength reached around 640 kPa, indicating the formation of a highly crosslinked and mechanically robust network.

The markedly higher strength of Red Angico foams suggests that the chemical functionality and molecular architecture of polyols derived from liquefied biomass strongly influence network density and stress transfer efficiency. However, the large variability observed at higher isocyanate indexes, reflected by the error bars, indicates that excessive NCO content can lead to heterogeneous microstructures, such as irregular cell size distribution or localized over-crosslinking, which compromises the uniformity of the foam structure. These results demonstrate that, although increasing the isocyanate index improves mechanical performance, an optimal range must be established to balance strength and structural homogeneity [46,47].

Overall, these results demonstrate that increasing the isocyanate index enhances compressive strength of liquefied Mahogany and Red Angico-based foams similarly to the presented before [24,48,49]. The significant variability observed across samples suggests that optimization of formulation and processing conditions will be critical for improving consistency and mechanical reliability in future applications.

The comparison between Mahogany and Red Angico foams becomes particularly meaningful when considering that the first four columns of each series, those at isocyanate indices 1.06, 1.49, 1.91, and 2.45 for Mahogany and 2.98, 3.83, 4.54, and 4.90 for Red Angico, were all produced using the same absolute amounts of isocyanate. The difference in isocyanate index stems from the differing OH values of the polyols: Mahogany has an OH value of 1200 mg KOH/g, while Red Angico’s is 600 mg KOH/g. Since the isocyanate index is based on the molar ratio of NCO groups to OH groups, Mahogany’s higher OH value means a lower isocyanate index for the same isocyanate input.

With equal isocyanate dosage (different OH value), Red Angico foams consistently exhibit higher compressive strength and modulus values across all corresponding points. This suggests that Red Angico’s lower OH content leads to a more isocyanate-rich system, favoring the formation of a stiffer, more crosslinked polymer network. In contrast, Mahogany, with more OH groups relative to the same amount of isocyanate, likely forms a network with lower crosslink density and more unreacted sites, resulting in softer foams.

The ideal OH value for polyurethane production has been reported to be between 300 and 800 and to significantly influence the physical and thermal properties of rigid polyurethane foams [50,51]. While properties like closed cell content, compression strength, and dimensional stability generally improve with increasing OH value due to higher crosslink density, other properties such as reaction times, density, and thermal conductivity show an optimal point around 500 OH value, indicating a complex interplay between mixture mobility and crosslinking reactions. The high OH value (≈1200 mg KOH/g) of liquefied Mahogany wood polyol can contribute to the relatively low compressive strength observed in these rigid polyurethane foams. OH value is known to be inversely related to the molecular weight of the polyol. A high OH number indicates a highly functional, short-chain polyol with numerous reactive OH groups, which in turn promotes very dense crosslinking in the polymer network. This results in a rigid, brittle foam structure that compromises the flexibility and performance needed for applications demanding cushioning and resilience [52]. Additionally, high OH value polyols are often highly viscous, which hinders effective mixing and uniform cell formation. Poor dispersion and rapid curing may lead to heterogeneous cell morphology, large cell size variance, and defects that impair strength. According to studies, this effect has been attributed to the excessive allophanate crosslinking between urethane linkages and residual isocyanate groups that increases the viscosity of the reacting mixture, which slows down both the gelling and foaming processes [50].

**Figure 5:**
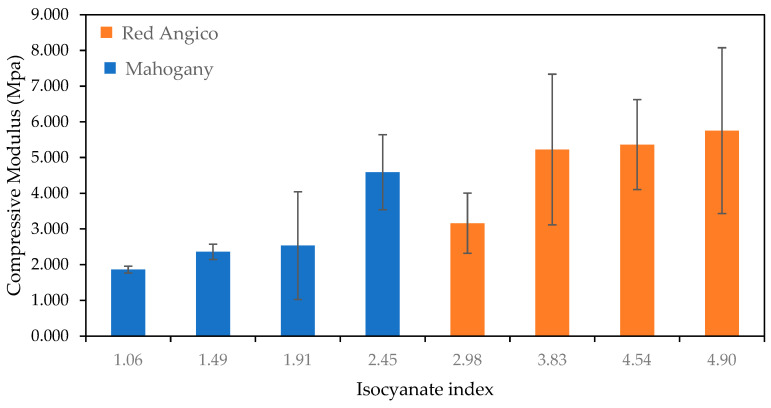
Variation in Compressive modulus with Isocyanate index for polyurethane foams derived from Mahogany (blue) and Red Angico (Orange). Error bars represent the standard deviation.

Figure 5 shows how the compressive modulus of foam varies with the isocyanate index for the two types of biomass: Mahogany (blue bars) and Red Angico (orange bars). Overall, the compressive modulus tends to increase with the isocyanate index for both materials, but the trends and performance levels differ between them. For Mahogany-based foams, there is a gradual increase in compressive modulus as the isocyanate index rises from 1.06 to 2.45, maxing out at around 4.5 MPa. The variability in the data, indicated by the error bars, increases notably at an index of 1.91, suggesting inconsistent structural integrity at this ratio. Starting at an index of 2.98, Red Angico-based foams also show a compressive modulus increase with the isocyanate index, and continue to increase steadily, reaching about 5.7 MPa at an index of 4.90. Despite the improved mechanical performance at higher isocyanate indices, the broad error margins again highlight the influence of microstructural variability on the reproducibility of compressive properties.

**Figure 6:**
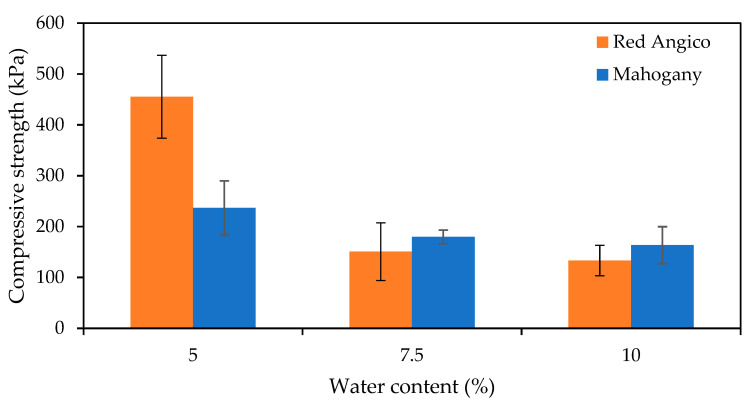
Variation in Compressive strength with water content for Mahogany (blue) and Red Angico (Orange). Error bars represent the standard deviation.

Figure 6 illustrates how the compressive strength of Mahogany and Angico-based polyurethane foams changes with varying water content, which acts as a chemical blowing agent. The trend shows a clear decrease in compressive strength as the water content increases from 5% to 10% and this decrease is observed for both polyols. This suggests that lower water levels lead to denser, more compact foam structures with stronger mechanical properties, but possibly also less consistency in cell structure. As water content increases, compressive strength decreases, which was expected because more water generates more CO_2_ during the reaction, resulting in higher porosity and lower foam density. Excessive water content can be detrimental, as it generates a negative pressure gradient due to the rapid diffusion of CO_2_ through the cell walls, leading to cell deformation [50].

In summary, increasing water content reduces the compressive strength of foams due to increased porosity and decreased density. A trade-off exists between lightness and mechanical integrity, and an optimal blowing agent level should balance both, depending on the intended application.

## 4. Conclusions

This study demonstrates that the chemical composition of tropical hardwood species plays a decisive role in biomass liquefaction efficiency, polyol functionality, and the resulting mechanical performance of rigid polyurethane foams. Red Angico (*Anadenanthera colubrina*) and Mahogany (*Swietenia macrophylla*) exhibited markedly different behaviors throughout the liquefaction and foam production stages, highlighting the importance of species-specific processing strategies for bio-based polyurethane systems.

Mahogany, characterized by its high hemicellulose content, showed superior liquefaction efficiency, achieving up to 93.4% conversion at 180 °C after 60 min. This behavior reflects the higher susceptibility of hemicelluloses to acid-catalyzed depolymerization, resulting in polyols with very high hydroxyl values (≈1200 mg KOH/g). However, despite the high degree of liquefaction and OH functionality, the resulting polyols led to polyurethane foams with comparatively lower compressive strength and modulus. This outcome is attributed to excessive polyol reactivity and viscosity, which likely impaired foam cell homogeneity and promoted overly brittle polymer networks.

In contrast, Red Angico exhibited lower liquefaction yields under the same conditions, consistent with its higher cellulose and lignin contents, which confer greater resistance to acid hydrolysis. Nevertheless, the polyols obtained from Red Angico presented moderate hydroxyl values (≈600 mg KOH/g), falling within the optimal range reported for rigid polyurethane applications. As a result, Red Angico-based foams displayed significantly superior compressive strength and modulus when produced with equivalent isocyanate amounts, indicating a more favorable balance between crosslink density, network formation, and cellular structure.

The mechanical performance of the foams was strongly influenced by the formulation parameters. Increasing the isocyanate index raised both the strength and the compression modulus for both biomass-derived polyols, confirming the critical role of crosslink density in the performance of rigid foams. On the other hand, increasing the water content led to a systematic reduction in compressive strength due to higher porosity and lower apparent density, emphasizing the need to optimize blowing agent levels according to the intended application.

Overall, these results demonstrate that high liquefaction yield and high hydroxyl value do not necessarily translate into superior polyurethane foam performance. Instead, an appropriate balance between biomass composition, liquefaction conditions, and polyol functionality is essential. The findings highlight Red Angico as a particularly promising, yet underexplored, renewable feedstock for the production of high-performance bio-based polyurethane foams, while Mahogany-derived polyols may require additional formulation or processing adjustments to fully exploit their high reactivity.

This work provides new insights into the structure–process–property relationships governing lignocellulosic biomass liquefaction and polyurethane foam formation. Future studies should focus on refining liquefaction conditions, controlling recondensation reactions, and tailoring polyol molecular architecture to further enhance foam uniformity, durability, and scalability, contributing to the development of sustainable alternatives to petroleum-based polyurethane materials.

## Figures and Tables

**Table 1:** Parameters chosen for polyurethane foams based on Red Angico and Mahogany polyols.

Polyol	pMDI	Water (Blowing Agent)	Tegostab (Surfactant)
3 g	1.0–4.9	5–10%	3%

**Table 2:** Chemical composition of Red Angico and Mahogany woods.

Parameters	Red Angico	Mahogany
	%	%
DCM extractives	0.22	0.42
EtOH extractives	3.25	4.02
Hot water	1.77	1.70
Klason Lignin (Total)	20.63	19.51
α-Cellulose	48.44	18.24
Hemicelluloses	25.68	56.11

DCM (dichloromethane); EtOH (ethanol).

## Data Availability

The original contributions presented in this study are included in the article. Further inquiries can be directed to the corresponding author.
